# Evaluation of Osteoprotegerin (OPG) and Receptor Activator of Nuclear Factor Kappa‐B Ligand (RANKL) Biomarker Concentrations in the Saliva of Patients with Periodontitis and Depression

**DOI:** 10.1002/cre2.70379

**Published:** 2026-05-17

**Authors:** Azadeh Esmaeilnejad, Ardeshir Lafzi, Seyedeh Morvarid Neishabouri, Mohammad Mohsen Bigham

**Affiliations:** ^1^ Department of Periodontics, School of Dentistry Shahid Beheshti University of Medical Sciences Tehran Iran; ^2^ Department of Psychiatry Kermanshah University of Medical Science Kermanshah Iran; ^3^ School of Dentistry Shahid Beheshti University of Medical Sciences Tehran Iran

**Keywords:** biomarker, depression, OPG, periodontitis, RANKL, saliva

## Abstract

**Background and Objectives:**

Periodontitis is a chronic inflammatory disease affecting tooth‐supporting structures, while depression is a common mental disorder with emotional and behavioral disturbances. Previous studies suggest a potential association between the two conditions. This study aimed to evaluate salivary concentrations of Receptor Activator of Nuclear Factor Kappa‐B Ligand (RANKL) and Osteoprotegerin (OPG)—key regulators of osteoclast activity and periodontal bone resorption—in patients with periodontitis and depression.

**Materials and Methods:**

In this cross‐sectional study, 80 patients from Shahid Beheshti University Dental School were categorized into four groups based on the presence or absence of periodontitis and depression. Periodontitis was defined as having ≥ 2 teeth in ≥ 2 quadrants with bleeding on probing, pocket depth > 3 mm, and interdental clinical attachment loss ≥ 1 mm. Depression was assessed using the Persian PHQ‐9, and individuals meeting criteria for major depressive disorder(MDD) were classified as depressed. Unstimulated saliva samples (1 mL) were collected, stored at −20°C, and analyzed for OPG and RANKL using ELISA kits. Statistical analyses included two‐way ANOVA (SPSS v27; *p* < 0.05 significant).

**Results:**

Among 80 participants (43 females, 37 males; mean age 45.3 years), RANKL, OPG, and the RANKL/OPG ratio were higher in depressed individuals, but differences were not statistically significant (*p* > 0.05). RANKL levels and the RANKL/OPG ratio were significantly higher in patients with periodontitis and increased with disease severity (*p* ≤ 0.05), while OPG showed no significant change.

**Conclusion:**

Periodontitis and its severity significantly influence salivary RANKL and the RANKL/OPG ratio, while depression does not. Within the limits of this cross‐sectional design, no evidence was found that RANKL and OPG act as intermediary biomarkers linking depression and periodontitis.

## 1 Introduction

Depression is a common mental disorder with multifactorial origins and both genetic and non‐genetic components (Sundararajan et al. 2015; Levinson 2006; Tiemeier 2003). Based on specific symptoms, depressive disorders are classified into major depressive disorder, persistent depressive disorders, and specified or unspecified depressive disorders (American Psychiatric Association and American Psychiatric Association 2013). Globally, depression is highly prevalent and often coexists with systemic conditions such as diabetes, cardiovascular disease, and cancer (Liu et al. 2020; Warren et al. 2014; Lawrence et al. 2013). Numerous studies have reported that many individuals with depression exhibit changes in immune‐inflammatory responses, characterized by elevated pro‐inflammatory cytokines, increased oxidative and nitrosative stress, altered tryptophan metabolism, and diminished levels of neurotrophic factors (Mac Giollabhui et al. 2021; Osimo et al. 2019). demonstrated a positive association between anhedonia‐like behavior and the RANKL/OPG ratio in mice, suggesting a potential mechanistic link relevant to human research (Zhang et al. 2020).

Periodontal disease is a broad term for a range of inflammatory diseases affecting the periodontium, which includes a set of structures supporting the teeth: the gums, cementum, periodontal fibers, and alveolar bone (de Molon et al. 2018). Periodontitis is diagnosed based on clinical and radiographic evidence of inflammation and attachment loss (Periodontitis et al. 2015). In the 2017 classification, periodontitis is staged according to severity and graded according to progression (Tonetti et al. 2018). The presence of pathogenic anaerobic microorganisms, plaque biofilm, and host immune responses are considered primary factors for this disease (Socransky 1970; Gurenlian 2007). At the histological level, periodontal disease leads to inflammatory infiltration consisting of neutrophils and leukocytes, which activate immune cells such as lymphocytes to release prostaglandins, interleukin‐1β (IL‐1β), tumor necrosis factor‐α (TNF‐α), interleukin‐6 (IL‐6), RANKL, and OPG (de Molon et al. 2018; Mundy 1991). RANKL, also known as TNFSF11, is the eleventh member of the OPG ligand family, expressed by osteoblasts, stromal cells, fibroblasts, B cells, and T cells when stimulated by cytokines and bacterial lipopolysaccharides. Its action is mediated by binding to RANK on the surface of pre‐osteoclast/osteoblast cells, leading to increased osteoclast activity (Dutzan et al. 2009; Cochran 2008). OPG, also known as osteoclastogenesis inhibitory factor or TNFRSF11B, is a soluble circulating RANKL receptor that antagonizes RANK‐RANKL interaction, thereby promoting bone formation by inhibiting osteoclastogenesis (Bartold et al. 2010). RANKL and OPG regulate bone resorption by positively or negatively stimulating RANK on osteoclast cells (Kadkhodazadeh et al. 2013). Furthermore, studies have shown that the RANKL/OPG ratio is higher in periodontitis‐affected sites compared to healthy sites (Kadkhodazadeh et al. 2013) and systemic conditions such as diabetes and smoking have also been associated with elevated ratios (Ali et al. 2019; Polak and Shapira 2018; Behfarnia et al. 2016).

The impact of depression on oral health, whether through diminished hygiene or biological mechanisms, has been widely investigated and evidence indicates that depression and stress alter immune responses, increasing susceptibility to adverse conditions and potentially compromising periodontal health (Sundararajan et al. 2015; Biondi and Zannino 1997). The link between depression and periodontitis is thought to be mediated by factors such as cortisol in saliva and blood, lipopolysaccharides, and other markers of systemic inflammation and cellular stress (Martínez et al. 2022). Psychological stress triggers immune responses that may promote the onset and progression of periodontal disease (Nolde et al. 2022). Numerous studies have demonstrated a bidirectional relationship between mental health disorders and oral inflammatory diseases (Aldosari et al. 2020; Choi et al. 2022; Zheng et al. 2021). However, heterogeneity in study populations—regarding age, gender, disease severity, smoking, alcohol use, and occupation—has led to inconsistent findings in population‐based research (Zheng et al. 2021).

Given the influence of psychological factors on chronic disease progression, further studies are required to clarify the association between depression and periodontitis. Most prior research has focused on biomarkers altered in either depression or periodontitis separately, with limited emphasis on their interaction. While markers such as cortisol (Rahate et al. 2022) and IL‐6 (Rodríguez Franco et al. 2020)have been studied extensively, OPG and RANKL remain underexplored. Salivary biomarkers such as RANKL and OPG may offer non‐invasive indicators of periodontal bone metabolism with potential translational relevance. Few studies have simultaneously assessed these biomarkers in both conditions, leaving a gap in understanding their potential interaction. This study therefore aimed to evaluate salivary concentrations of RANKL and OPG in patients with and without periodontitis and depression. The null hypothesis was that salivary RANKL and OPG concentrations would not differ according to depression or periodontitis status.

## 2 Method and Material

### 2.1 Study Design and Ethical Considerations

In this descriptive cross‐sectional study, sampling was performed randomly among patients attending Shahid Beheshti Medical University School of Dentistry, Tehran, Iran, from 2022 to 2023. The study protocol was reviewed and approved by the Ethics Committee of Shahid Beheshti University of Medical Sciences (approval code: IR. SBMU. DRC. REC.1403.122). All procedures involving human participants were conducted in accordance with the ethical standards of the institutional research committee and with the 1964 Helsinki Declaration and its later amendments. All participants provided written informed consent prior to inclusion in the study.

#### 2.1.1 Participants and Sample Size

Eighty participants were enrolled and categorized into four groups (*n* = 20 per group) based on the presence or absence of periodontitis and depression:

1. Healthy controls
2. Depression without periodontitis
3. Periodontitis without depression
4. Periodontitis with depression

All participants were systemically healthy (ASA I–II classification) and aged 35–65 years. The sample size (n = 20 per group) was determined based on a previous similar study (Behfarnia et al. 2016) and a power analysis (α = 0.05, β = 0.2, power = 80%).

Participants were randomly selected from patients attending Shahid Beheshti Medical University School of Dentistry, Tehran, Iran, from 2022 to 2023. Participants were recruited consecutively from patients attending Shahid Beheshti University Dental School over a 4‐month period. Eligibility was assessed based on predefined inclusion and exclusion criteria. Participants were assigned to one of the four study groups according to their periodontal status and depression assessment at the time of examination. Inclusion criteria included willingness to participate, presence of at least 20 teeth, and fulfillment of diagnostic criteria for either periodontitis or depression (or both). Exclusion criteria were pregnancy, lactation, smoking, alcohol consumption, use of any medications such as antibiotics or non‐steroidal anti‐inflammatory drugs (NSAIDs) during the previous 3 months, and a history of systemic diseases (e.g., diabetes, cardiovascular disease) or major post‐traumatic events (e.g., bereavement, serious accident).

All participants who met the inclusion criteria and did not meet exclusion criteria were initially informed about the study objectives and procedures. Those who agreed were given a detailed consent form to review and sign. Demographic data were then recorded. The PHQ‐9 questionnaire, which included items on demographic information, medical history, medication use, smoking status, and recent traumatic events, was administered verbally, with responses referring to the previous 2 weeks. Subsequently, an intraoral examination using a periodontal probe was conducted to diagnose the presence and severity of periodontitis, and findings were documented. Based on the questionnaire and clinical examination, participants were assigned to one of four study groups.

**Figure 1:**
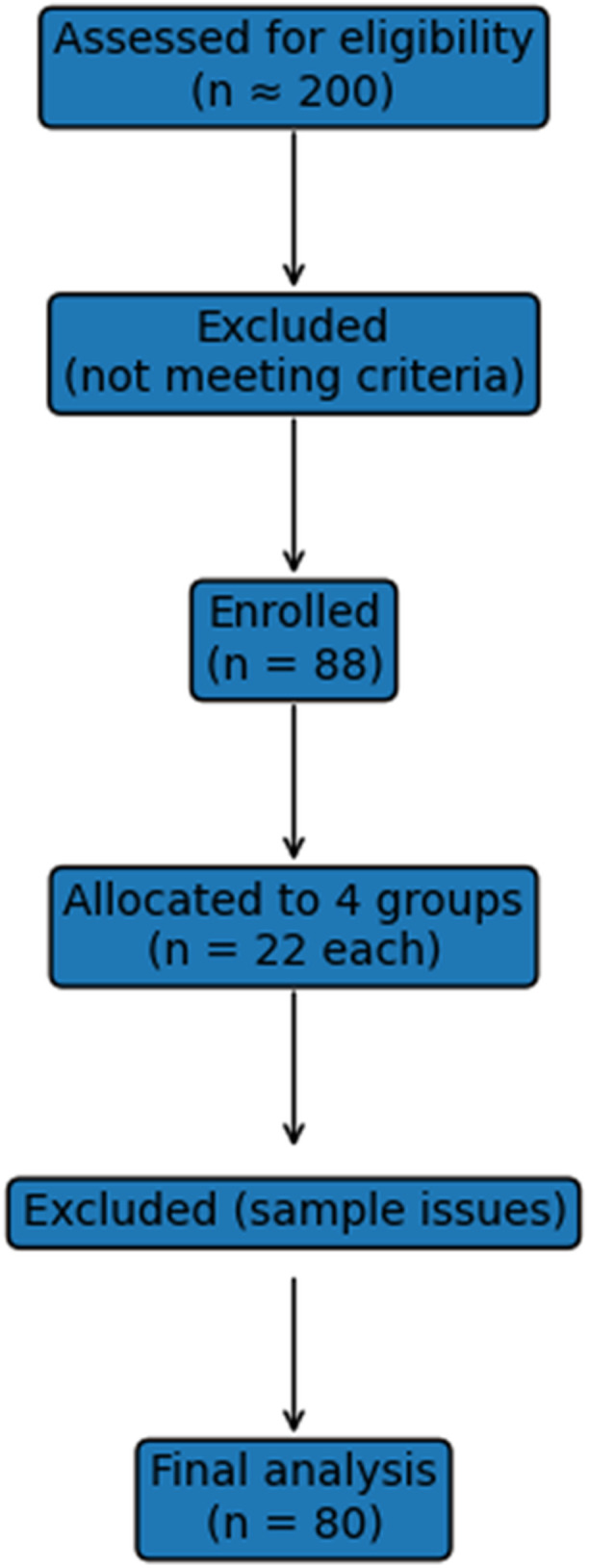
Participant flow diagram.

The process of participant recruitment, exclusion, and group allocation is illustrated in Figure 1.

### 2.2 Depression Assessment

The Persian version of the 9‐item Patient Health Questionnaire (PHQ‐9) was used to diagnose depression in this study. Recently, the 9‐item Patient Health Questionnaire (PHQ‐9) has been identified as the most reliable tool for screening for depression (Levis et al. 2020; El‐Den et al. 2018). The Patient Health Questionnaire (PHQ) is a new tool for diagnosing depression and other mental disorders based on criteria, and is commonly used in primary care. Although the PHQ‐9 has half the number of questions (9 items) compared to many other depression questionnaires, it has comparable sensitivity and specificity. It includes the 9 actual criteria based on which DSM‐IV diagnoses of depressive disorders are made (Kroenke et al. 2001). Major depressive disorder (MDD) is diagnosed if 5 or more of the 9 criteria for depressive symptoms have been present “more than half the days” in the past 2 weeks, and one of the symptoms is depressed mood or anhedonia. One of the 9 symptom criteria (“thoughts that you would be better off dead or of hurting yourself in some way”) is important if present, regardless of duration. Before a final diagnosis, the physician is expected to rule out physical causes of depression, normal grief, and a history of a manic episode (Kroenke et al. 2001). The Persian version of the PHQ‐9 is a valid and reliable tool for diagnosing major depressive episodes in clinical settings, and is also useful for diagnosing major depressive episodes (MDE) (Ardestani et al. 2019; Dadfar et al. 2018). Considering these advantages, as well as the brevity and conciseness of this questionnaire, which is desirable for the patient, the Persian version of the Patient Health Questionnaire‐9, or PHQ‐9 for short, was used to diagnose depression in this study. The questionnaire was self‐administered under supervision, and the results were interpreted under the supervision of a psychiatrist based on PHQ‐9 scores rather than a clinical psychiatric diagnosis.

Therefore, depression was assessed using the Persian version of the Patient Health Questionnaire‐9 (PHQ‐9). The questionnaire was self‐administered by participants. Classification of depression was based on the DSM‐IV–based diagnostic algorithm for major depressive disorder, requiring the presence of at least five symptoms, including either depressed mood or anhedonia.

### 2.3 Periodontal Examination

Periodontal health was defined as the absence of interdental clinical attachment loss (CAL) and probing pocket depth ≤ 3 mm. Individuals with gingivitis, characterized by bleeding on probing without attachment loss, were included in the non‐periodontitis control group. Individuals with periodontitis were defined as having at least two teeth in at least 2 quadrants with BOP, PD > 3 mm, and CAL > = 1 mm (Periodontitis et al. 2015; Papapanou et al. 2018; Armitage 1999). The stage or severity of the disease was determined based on the amount of CAL: CAL between 1 and 2 in Stage I, between 3 and 4 in Stage II, and 5 or greater than 5 in Stages III and IV (Tonetti et al. 2018).

### 2.4 Saliva Collection and Biochemical Analysis

Samples were collected after the initial diagnosis and before the start of any treatment. Unstimulated saliva was obtained in the morning, approximately 1 h after fasting (except for water intake). Participants were instructed to allow saliva to accumulate in the floor of the mouth and then expectorate approximately 1 mL into a sterile 15 mL tube. Samples contaminated with blood were discarded, and new samples were collected. After collection, samples were kept in a household refrigerator for 30 min and subsequently frozen at −20°C or lower until analysis.

Prior to analysis, all samples were centrifuged at 3000 rpm for 10 min. According to the manufacturer's protocol, saliva samples were used directly without any dilution after centrifugation and removal of debris.

Salivary concentrations of RANKL and OPG were measured using enzyme‐linked immunosorbent assay (ELISA) kits (Padgin Teb Co., PTC, Iran; Cat. No. PTC‐10620‐H9648 for RANKL and PTC‐11558‐H9648 for OPG) according to the manufacturer's instructions. The assay range for RANKL was 30–960 pg/mL with a sensitivity of 4 pg/mL, and for OPG was 50–1600 pg/mL with a sensitivity of 6 pg/mL. Standard serial dilutions supplied in the kit were used to construct the calibration curves.

Kits were stored at 2°C–8°C until use, and all reagents and samples were equilibrated to room temperature (approximately 23°C) 30 min before the assay. The procedure included preparing the standard solutions, adding standards and saliva samples to the wells, preparing the washing solution at a ratio of 1:30 (30×), washing, adding the chromogen solutions, stopping the reaction, and reading the absorbance at 450 nm using a microplate reader. Standard curves were plotted for each biomarker to determine concentrations.

### 2.5 Statistical Analysis

Data were analyzed using SPSS software (version 27.0; IBM Corp., Armonk, NY, USA). Two‐way ANOVA was used to compare biomarker concentrations between groups, and robust tests were applied to assess the association between the severity of periodontitis and biomarker concentrations. A *p*‐value < 0.05 was considered statistically significant.

## 3 Results

As described in Materials and Methods section, participants were categorized into four groups:

1. Healthy controls
2. Depression without periodontitis
3. Periodontitis without depression
4. Periodontitis with depression

This study included a total of 80 participants, with 43 females (53.75%) and 37 males (46.25%). The average age of the participants was 45.3 years, ranging from 35 to 65 years. The distribution of gender and age in each of the 4 groups is visible in the Table 1.

**Table 1:** Age and gender distribution of participants in each group.

Descriptive information				
	Age		Sex	
Group	*N*	Mean	Female(*N*,%)	Male(*N*,%)
1.00	20	43.20	11(55%)	9(45%)
2.00	20	43.55	14(70%)	6(30%)
3.00	20	48.90	8(40%)	12(60%)
4.00	20	45.80	10(50%)	10(50%)
Total	80	45.36	43(53.75%)	37(46.25%)

### 3.1 Biomarker Concentrations Across Groups

The mean concentrations of OPG and RANKL biomarkers, and the RANKL/OPG ratio, for each of the four study groups are presented in the Table 2.

**Table 2:** Mean biomarker concentrations (pg/mL) and ratios across the four study groups.

Periodontitis	Depression	RANKL (Mean ± SD) (pg/mL)	OPG (Mean ± SD) (pg/mL)	RANKL/OPG (Mean ± SD)	*N*
No	No	78.75 ± 35.38	203.46 ± 69.45	0.38 ± 0.12	20
No	Yes	95.85 ± 48.70	191.26 ± 53.08	0.47 ± 0.15	20
Yes	No	110.08 ± 44.92	192.73 ± 65.80	0.57 ± 0.16	20
Yes	Yes	130.05 ± 76.71	216.96 ± 84.19	0.59 ± 0.21	20
Without periodontitis		87.30 ± 42.90	197.36 ± 61.32	0.42 ± 0.14	40
With periodontitis		120.06 ± 62.86	204.84 ± 75.58	0.58 ± 0.18	40
Without depression		94.41 ± 42.95	198.09 ± 67.00	0.47 ± 0.17	40
With depression		112.95 ± 65.74	204.11 ± 70.67	0.53 ± 0.19	40
Total	Total	103.66 ± 55.95	201.10 ± 68.49	0.50 ± 0.18	80

### 3.2 Overall Biomarker Analysis

Table 3 summarizes the impact of periodontitis and depression on key biomarkers (RANKL/OPG, RANKL, and OPG) by Two‐way ANOVA analysis. The p‐values indicate whether the effects are statistically significant.

**Table 3:** Effect of diseases on biomarker levels and their ratios.

Independent variable	RANKL/OPG (*p v*alue)	RANKL (*p* value)	OPG (*p v*alue)
Periodontitis	< 0.001	0.008	0.629
Depression	0.123	0.127	0.698

### 3.3 Periodontitis Severity

Table 4 shows the number of participants and the mean salivary concentrations (Mean ± SD) of the evaluated biomarkers (RANKL, OPG, and RANKL/OPG ratio) in each stage of periodontitis.

**Table 4:** Comparison of biomarker concentrations among different stage of periodontitis.

Stage	*N*	OPG (Mean ± SD) (pg/mL)	RANKL (Mean ± SD) (pg/mL)	RANKL/OPG (Mean ± SD) (pg/mL)
1	14	179.16 ± 69.87	72.78 ± 24.60	0.41 ± 0.08
2	18	212.20 ± 56.31	125.16 ± 35.07	0.59 ± 0.04
3	8	233.23 ± 112.63	191.34 ± 86.96	0.85 ± 0.19
Total	40	204.84 ± 75.58	120.06 ± 62.86	0.58 ± 0.18

**Chart 1:**
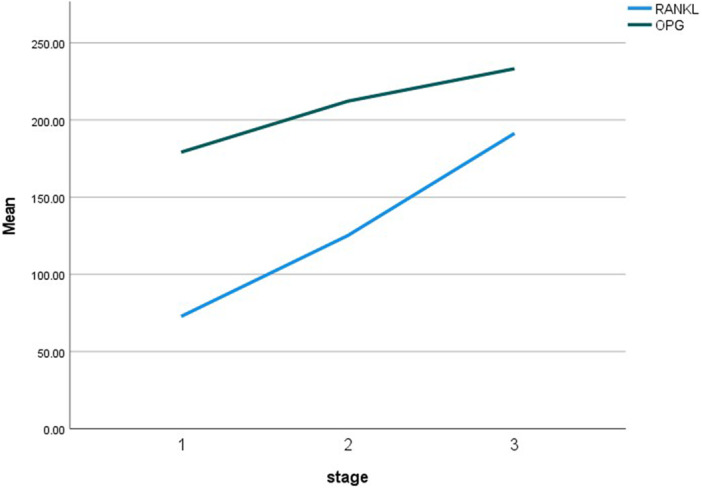
Mean OPG and RANKL concentrations (pg/mL) by periodontitis stage.

**Chart 2:**
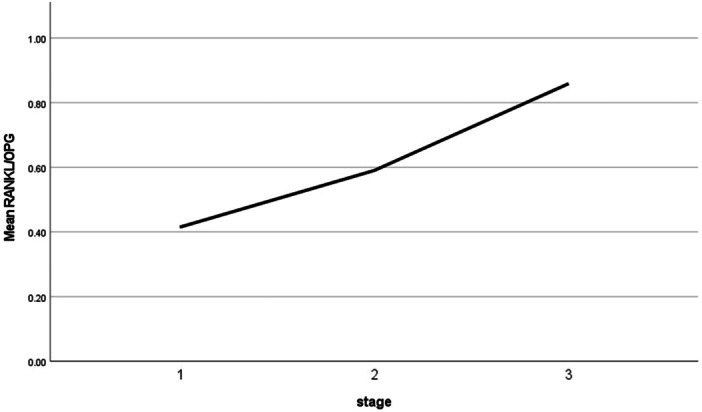
Mean RANKL/OPG ratio by periodontitis stage.

As shown in Charts 1 and 2, which illustrate the mean concentrations of OPG and RANKL biomarkers (in pg/mL) and their ratio based on the severity (stage) of periodontitis, we observed that with increasing periodontitis severity, the concentrations of RANKL and OPG, as well as the RANKL/OPG ratio, increased.

RANKL concentration and the RANKL/OPG ratio were significantly associated with periodontitis severity (*p*‐value ≤ 0.05), indicating an increase in both with increasing severity of periodontitis. Although OPG concentration increased with periodontitis severity, this association was not statistically significant (*p*‐value > 0.05). (Table 5).

**Table 5:** Association between periodontitis severity and biomarker concentrations and ratios.

		Sig.
OPG	Between different stage of periodontitis	0.238
RANKL	Between different stage of periodontitis	0 > 0.001
RANKL/OPG	Between different stage of periodontitis	0 > 0.001

## 4 Discussion

The initial hypothesis that OPG and RANKL could act as intermediary biomarkers linking periodontitis and depression was not supported by the results of this study.

Overall, our biomarker analysis revealed no significant difference in mean OPG levels between individuals with and without periodontitis or depression. However, RANKL levels and the RANKL/OPG ratio were significantly higher in individuals with periodontitis and increased with disease severity, while no significant difference was found based on depression status.

Given evidence linking psychological factors, particularly depression, to chronic diseases (Decker et al. 2021; Nascimento et al. 2019; Liu et al. 2018), and the limited focus on OPG and RANKL in this context, our study aimed to explore whether these biomarkers could serve as a biological link between periodontitis and depression. Through a descriptive‐analytical, cross‐sectional design, we sought to address this gap and assess the potential role of inflammation in connecting these two conditions.

We first evaluated the effect of periodontitis and its severity on the salivary biomarkers. Our findings are consistent with several previous studies demonstrating elevated salivary RANKL levels and an increased RANKL/OPG ratio in patients with periodontitis, while changes in OPG levels remain controversial. For instance, Teodorescu et al. and Asif et al. reported significant increases in RANKL and the RANKL/OPG ratio in periodontitis patients, with no significant differences in OPG levels (Teodorescu et al. 2019; Asif et al. 2021). In contrast, other investigations, such as those by Abdullameer et al. and Bostanci et al. observed significant alterations in OPG, though such discrepancies may be attributable to differences in methodology, including the use of gingival crevicular fluid (GCF) instead of saliva and the separation of gingivitis as a distinct group (Abdullameer and Abdulkareem 2023; Bostanci et al. 2007). In our study, individuals with gingivitis were included in the control group, which may have reduced the mean OPG levels in this group (Abdullameer and Abdulkareem 2023; Bostanci et al. 2007), thereby diminishing the observed differences between the control and periodontitis groups.

Regarding disease severity, we employed the contemporary classification system based on staging and grading, which provides a more comprehensive assessment than the previously used 1999 classification (Tonetti et al. 2018; Armitage 1999). Some studies support our findings, indicating a positive association between disease severity and both salivary RANKL concentrations and the RANKL/OPG ratio. For example, Teodorescu et al. reported such correlations in patients with chronic and aggressive periodontitis (Teodorescu et al. 2019). Conversely, other studies, such as those by Lu et al. and Sakellari et al. did not observe significant associations between biomarker levels and disease severity (Lu et al. 2006; Sakellari et al. 2008). Interestingly, findings from studies by Ochanji et al. and Bostanci et al. reported that salivary RANKL levels increased and OPG levels decreased with advancing periodontal disease, supporting their potential utility as adjunctive diagnostic markers (Bostanci et al. 2007; Ochanji et al. 2017). The variability in findings across studies may reflect differences in classification criteria, group definitions, and sampling techniques.

Next, we explored the effect of depression on salivary OPG, RANKL, and their ratio. Few studies have directly examined this relationship, often focusing on antidepressant effects instead. Lee et al. (2024) found that higher plasma RANKL was linked to fewer depressive symptoms in hemodialysis patients, while OPG showed no association (Lee et al. 2024). This partly agrees with our OPG results but differs for RANKL, likely due to differences in populations, depression assessments, and sample types. Oteo‐Álvaro Á et al. (2023) reported that Denosumab, an anti‐RANKL antibody, may cause neurological symptoms through effects on neuroinflammation and depression (Oteo‐Álvaro et al. 2023). In animals, Zhang et al. (2020) showed a positive correlation between RANKL/OPG ratio and depression‐like behavior, suggesting anti‐RANKL treatments might help (Zhang et al. 2020). These studies show conflicting results: human studies suggest higher RANKL reduces depression symptoms, while animal data suggest the opposite. The discrepancy between human and animal studies may be attributed to differences in biological matrices (saliva *vs.* plasma or serum), study populations with underlying systemic conditions, and variations in the conceptualization and assessment of depression. Differences in study subjects and methods make firm conclusions difficult, but they collectively highlight the need for further research on depression and OPG/RANKL biomarkers.

Finally, we assessed whether OPG and RANKL could mediate the relationship between periodontitis and depression. A systematic review by Neupane et al. (2022) highlighted inconsistent findings in human studies regarding biomarkers linking these conditions, despite evidence from animal models supporting immune‐inflammatory involvement (Neupane et al. 2022). Most research has focused on cortisol and interleukins, with limited investigation of OPG and RANKL, revealing a significant gap addressed by our study. Sampling methods varied across studies, including gingival crevicular fluid, blood, and saliva.

Our study employed a descriptive‐analytical, cross‐sectional design, consistent with similar research examining biomarker concentrations across groups. To specifically attribute variations in OPG, RANKL, and their ratio to periodontitis and depression, we rigorously controlled for confounding factors. Gender and age distributions were balanced across groups, with no significant differences observed despite a higher proportion of females in the depression cohorts, reflecting known epidemiological trends (Parker and Brotchie 2010). The mean age of 47.4 years was comparable among groups, mitigating age‐related confounding given its established association with periodontitis progression (Eke et al. 2015) and bone resorption mediated by RANKL and the RANKL/OPG ratio (Bartold et al. 2010; Kadkhodazadeh et al. 2013; Boyce and Xing 2007). Additional confounders, including smoking, systemic diseases, and medication use, were addressed by excluding smokers and individuals on drugs affecting bone metabolism or inflammation (Ali et al. 2019; Polak and Shapira 2018; Behfarnia et al. 2016; Toffoli et al. 2016).

While oral hygiene behaviors could not be standardized and thus remain a potential confounder, this limitation is inherent in studies involving depressed and periodontitis‐affected populations. Oral hygiene status was not objectively measured using indices such as the Plaque Index or Oral Hygiene Index. This may represent a potential confounding factor, as oral hygiene practices can influence periodontal inflammation and salivary biomarker levels. Furthermore, the cross‐sectional design precludes causal inference, and the relatively small sample size may have reduced statistical power. Other unmeasured variables, such as socioeconomic status and dietary habits, could also have influenced biomarker levels.

A major difference in depression‐related studies lies in diagnostic tools. While Lee D‐Y et al. used the BDI (Lee et al. 2024), we employed PHQ‐9, a shorter yet DSM‐IV–aligned instrument with comparable diagnostic accuracy (Kroenke et al. 2001). Although tools with broader scope or higher cut‐off scores (e.g., PHQ‐9 ≥ 15) may offer greater sensitivity, we selected PHQ‐9 for its brevity, diagnostic validity, and proven reliability in Persian (Kroenke et al. 2001; Ardestani et al. 2019; Dadfar et al. 2018; Spitzer and Group PHQPCS, Group PHQPCS 1999), ensuring practicality during clinical visits.

To address the variability and inconsistencies observed across studies, future research should involve larger and more diverse populations, clearly distinguish gingivitis from periodontal health, and apply the latest classification criteria. Longitudinal and interventional studies are needed to better understand causal relationships. Furthermore, exploring the clinical relevance of RANKL inhibition and its potential influence on neuroinflammatory pathways may provide novel insights into the biological interplay between periodontitis and depression.

Although numerous studies have demonstrated associations between affective disorders and periodontitis (Decker et al. 2021; Nascimento et al. 2019; Liu et al. 2018), our study investigated this relationship from a biological perspective using OPG and RANKL. The findings indicate that while periodontitis significantly influences these biomarkers, depression does not. Within the limitations of this design, OPG and RANKL do not appear to act as intermediary biomarkers linking periodontitis and depression.

## 5 Conclusion

This study evaluated salivary OPG and RANKL concentrations as potential intermediaries between periodontitis and depression. The findings showed that periodontitis and its severity significantly affected RANKL levels and the RANKL/OPG ratio, while OPG levels did not change significantly. Depression had no significant impact on either biomarker. Therefore, within the limitations of this cross‐sectional study, RANKL and OPG cannot be considered biological mediators linking periodontitis and depression.

## Author Contributions

Azadeh Esmaeilnejad contributed to study design and manuscript writing. Ardeshir Lafzi contributed to supervision and data verification. Seyedeh Morvarid Neishabouri contributed to study design in the depression field and supervision. Mohammad Mohsen Bigham contributed to study execution, data collection, data analysis, and manuscript writing. All authors read and approved the final manuscript.

## Conflicts of Interest

The authors declare no conflicts of interest.

## Data Availability

The data that support the findings of this study are available from the corresponding author upon reasonable request.
